# The New Consumer Protection Act Controversy: Does It Apply to Doctors in India?

**DOI:** 10.7759/cureus.57538

**Published:** 2024-04-03

**Authors:** Satvik N Pai, Sankalp Yadav, Naveen Jeyaraman, Madhan Jeyaraman

**Affiliations:** 1 Orthopaedics, People's Education Society (PES) University Institute of Medical Sciences & Research, Bengaluru, IND; 2 Medicine, Shri Madan Lal Khurana Chest Clinic, New Delhi, IND; 3 Orthopaedics, ACS Medical College & Hospital, Dr. M.G.R. Educational and Research Institute, Chennai, IND; 4 Clinical Research, Virginia Tech India, Dr. M.G.R. Educational and Research Institute, Chennai, IND

**Keywords:** hospitals, doctors, medical services, cpa, consumer protection act

## Abstract

A new Consumer Protection Act (CPA) was introduced in 2019 and has created quite a stir in the medical fraternity. There is widespread uncertainty as to whether this new Act applies to the medical profession. However, on careful review of the original CPA of 1986, the definition of services included within the Act, and understanding the changes introduced in CPA 2019, the legal application of CPA 2019 to medical professionals becomes clear. While the term medical services has been removed from the list of services under the purview of the Act, the phrase “but not limited to” before listing the services leaves the door open for the inclusion of other services.

## Editorial

A new Consumer Protection Act (CPA) was introduced in 2019 and created quite a stir in the medical fraternity. There is widespread uncertainty as to whether this new Act applies to the medical profession. Several print media outlets were quick to publish poorly researched articles, which added to the controversy. To really understand the CPA, we need to understand the developments of this Act and the judgments of Indian courts in this matter from the beginning.

The original CPA of 1986

The CPA was first inducted into the Constitution of India in 1986 to provide for better protection of the interests of consumers. Its definition of a consumer included any person buying any goods or availing of any service for payment. The services listed as examples in Section 2(1)(o) were banking, financing insurance, transport, processing, supply of electrical or other energy, board or lodging or both, housing construction, entertainment, amusement, or the purveying of news or other information [1]. This did not explicitly mention doctors or medical services. However, the definition included a statement mentioning that the purview of the Act was not limited to the listed services. Patients were, however, not seen as “consumers,” and hence this Act was not involved in medical litigation for almost a decade since it was introduced.

It was only in 1995, in the judgment of the Indian Medical Association v. V.P. Shantha case, that the Supreme Court of India categorically stated that the medical profession would be brought under the CPA [2]. The only exceptions were doctors and hospitals, which provided all services free of charge to all patients. Doctors and hospitals rendering free as well as paid services could be charged under the Act even for the patients who were provided service free of charge. Following this, the CPA and Consumer Disputes Redressal Forums became additional avenues for litigation against medical professionals and saw a multitude of cases of alleged medical negligence. The Act underwent amendments in 1991, 1993, and 2002 but saw no change in relation to its applicability to medical services.

Doctors’ opinions

The majority of the medical community has long claimed that medical services should not be included in the CPA. The doctor-patient relationship is very different from one between a “consumer” and a “manufacturer or trader of goods.” It also has very few similarities to providing services like banking, transport, construction, etc., which are far more straightforward, predictable, and controllable. This led medical professionals to take up this matter on several occasions with lawmakers and elected government representatives since the beginning of the millennium. However, help was never forthcoming. The proposal for a new CPA in 2019 provided another opportunity. Several imminent doctors lobbied vehemently, requesting that medical services not be included in CPA and instead be guided by laws more appropriate for the medical profession.

The new CPA, 2019

A new CPA was introduced in 2019. In Section 2(42), the list of included services does not mention medical services [3]. This omission, despite the 1995 ruling of the honorable Supreme Court directing its inclusion, has been misconstrued by many as the exclusion of medical services from the purview of the Act [4,5]. However, this is not the case. The Act, in its definition of services, retains the phrase “but not limited to” before listing the services, thereby leaving the door open for the inclusion of other services. There is no statement in CPA 2019 explicitly excluding medical services from the Act. The mere removal of medical services from the list bears no legal significance. This should be understood by medical professionals in India, as the Consumer Disputes Redressal Forums will certainly continue to be an avenue for litigation against doctors and hospitals. All hope is, however, not lost. The discussions, appeals, and lobbying prior to the passing of CPA 2019 have brought the attention of the legal community and general public to this matter. The arguments of medical professionals have created awareness and raised legitimate questions about the suitability of the CPA for medical services. It has brought the medical community together to create a more united and organized attempt at bringing about a change in the law. We may have lost the battle, but we shall continue to fight the war.
